# Non-medical Use of Prescription Gabapentinoids (Gabapentin and Pregabalin) in Five European Countries

**DOI:** 10.3389/fpsyt.2021.676224

**Published:** 2021-04-28

**Authors:** Francina Fonseca, William Lenahan, Richard C. Dart, Esther Papaseit, Paul I. Dargan, David M. Wood, Marilena Guareschi, Icro Maremmani, Marc Auriacombe, Magí Farré, Norbert Scherbaum, Marta Torrens

**Affiliations:** ^1^Hospital del Mar, Institut de Neuropsiquiatria i Addiccions (INAD), Barcelona, Spain; ^2^Grup de Recerca en Addiccions, Institut Hospital del Mar d'Investigacions Mèdiques (IMIM), Barcelona, Spain; ^3^Department of Psychiatry and Legal Medicine, Universitat Autònoma de Barcelona (UAB), Cerdanyola del Vallès, Spain; ^4^Rocky Mountain Poison and Drug Safety, Denver Health and Hospital Authority, Denver, CO, United States; ^5^Clinical Pharmacology Unit, Hospital Universitari Germans Trias i Pujol (HUGTP-Germans Trias i Pujol Research Institute), Badalona, Spain; ^6^Department of Pharmacology, Therapeutics and Toxicology, Universitat Autònoma de Barcelona (UAB), Cerdanyola del Vallès, Spain; ^7^Clinical Toxicology, Guy's and St Thomas' NHS Foundation Trust, London, United Kingdom; ^8^Clinical Toxicology, Faculty of Life Sciences and Medicine, King's College London, London, United Kingdom; ^9^Association for the Application of Neuroscientific Knowledge to Social Aims (AU-CNS), Lucca, Italy; ^10^Vincent P. Dole Research Group, Santa Chiara University Hospital, University of Pisa, Pisa, Italy; ^11^Addiction Psychiatry Department, University of Bordeaux, Bordeaux, France; ^12^Addiction Team, Sanpsy Centre National de la Recherche Scientifique (CNRS) USR 3413, Bordeaux, France; ^13^Pôle Addictologie et Filière Régionale, Centre Hospitalier (CH) Charles Perrens and Centre Hospitalier Universitaire (CHU) de Bordeaux, Bordeaux, France; ^14^LVR-Hospital Essen, Department of Psychiatry and Psychotherapy, Medical Faculty, University of Duisburg-Essen, Essen, Germany

**Keywords:** gabapentin, pregabalin, non-medical use, prescription drugs, misuse

## Abstract

**Background:** Non-medical use (NMU) of prescription GABA analogs (pregabalin and gabapentin) has been reported especially in opiate dependent persons. However, by now the prevalence of NMU of gabapentinoids in the general population has not been sufficiently evaluated. The aim of this research paper is to determine the prevalence of prescription GABA analog NMU and associated demographics in five European countries with special detail of Spain.

**Methods:** The RADARS Survey of Non-Medical Use of Prescription Drugs Program (NMURx) is a harmonized series of contemporaneous cross-sectional surveys of adults conducted in multiple countries. NMURx collects data from the general population in each participating country about NMU of prescription drugs, illicit drugs, and associated demographics. NMU was defined as “using a medication without a doctor's prescription or for any reason other than what was recommended by their doctor.” Responses from Spain (4Q2017, *n*=10,062) were analyzed in detail. Comparative data were available from France, Germany, Italy, and UK. Responses were collected using non-probability quota sampling and post-stratification population weighting was applied to reflect the national distributions of adults, based on age, gender, and census region. Rates of NMU and associated demographics were reported as rate of past 90-day NMU per 100,000 adult population with 95% confidence intervals.

**Results:** Germany (1,197 per 100,000 adult population [95% CI: 1,004.3–1,379.1]) and United Kingdom (1,067 per 100,000 adult population [95% CI: 851.3–1,283.2]) presented the highest prevalence of gabapentinoids NMU. In Spain the prevalence of past 90 days GABA analog NMU was: 344.4, 95% (CI 204.8–484.0), with male predominance. Those who non-medically use GABA analogs had a higher prevalence of lifetime chronic pain, lifetime illicit drug use, and previous substance abuse treatment. In Spain, 20% of respondents who ever have used gabapentinoids, reported a lifetime NMU; the prevalence was higher for pregabalin 624 (6.2%) than for gabapentin 444 (4.4%). The main reasons for use were to self-treat pain and other medical conditions.

**Conclusions:** The risk of NMU of gabapentinoids should not be neglected. Subjects with a history of chronic pain and lifetime substance use disorders had an increased risk of NMU of gabapentinoids.

## Introduction

Gabapentinoids, pregabalin and gabapentin, are widely used for the treatment of neuropathic pain and epileptic disorders according to the United States (US) Food and Drug Administration (FDA). Both gabapentin and pregabalin have been approved by the European Medicine Agency (EMA) for neuropathic pain and generalized anxiety disorder, respectively. Additionally, some off-label uses of gabapentinoids include treatment for chronic lower back pain, insomnia, migraine, social phobia, panic disorder, mania, bipolar disorder, and alcohol withdrawal (1, 2).

Gabapentinoids are now among the most commonly prescribed medications in most countries (3). For instance, the overall rate of pregabalin prescriptions use increased from 1.0 per 1,000 individuals in 2013 to 22.0 per 1,000 individuals in 2014 in Ontario, Canada (4). Also, there has been a progressive increase in the reported cases of misuse and dependence to the European Medicines Agency's EudraVigilance database, specifically in subjects with previous history of substance use disorders (3).

At the pharmacological level, gabapentinoids selectively bind to the α2δ-subunit of voltage-gated calcium channels in central nervous system neuronal tissues. This in turn increases the GABA levels and decreases other excitatory neurotransmitters (5). This mechanism is associated with their antinociceptive, anticonvulsant, anxiolytic, and sleep-modulating effects (6). Gabapentinoids have significant risks despite their reputation as safe drugs. Sedation, dizziness, gait instability, and feeling of intoxication are quite common side effects; as many as one in three patients taking therapeutic doses experience dizziness or somnolence (7). Although, both substances share some mechanisms of action, they also have some pharmacokinetic differences that could explain differences in their abuse potential; for instance, pregabalin is absorbed more rapidly by oral route, with maximum plasma concentrations attained within 1 h, whereas, maximum plasma concentrations of gabapentin are detected 3–4 h after oral administration. Pregabalin absorption is linear, and gabapentin absorption is saturable (non-linear –zero-order- process) with less predictable pharmacokinetics. Bioavailability is also different; pregabalin has a 90% bioavailability independently from the dosage, but gabapentin bioavailability changes with dosage, from 60% at 900 mg/day to 33% at 3,600 mg/day. On the other hand, similarities in pharmacokinetics are: both can be given without regard of meals, they do not bind to plasma proteins and both are excreted renally with an elimination half-live of 6 h (7). The linear pharmacokinetics of pregabalin and its greater potency explains its steep dose-response relationship and differences in abuse potential and severe adverse events as respiratory depression.

Evidence regarding misuse and diversion of gabapentinoids has grown in recent years (8–10). The first description of their misuse and abuse were published in 2010 (11). Prevalence of misuse and abuse in the general population is an estimated 2.5% (12) but, the rates in people suffering a substance use disorder (SUD) is higher (pregabalin: 3–68%; gabapentin: 15–22%) (1). In a systematic review aimed to evaluate the abuse liability of gabapentin and pregabalin, the authors found that pregabalin had a greater potential for addiction than gabapentin based on the magnitude of behavioral dependence symptoms, transitions from prescription to self-administration, and the durability of the self-administrations (8).

Current research suggests that the addictive potential of gabapentinoids is primarily a concern among patients with other substance use disorders, especially opioid use disorder (8). The reasons that motivate gabapentinoid misuse and abuse are not clearly described. Also, the subjective effects described by people who report non-medical use are multiple: self-treatment of pain and other medical conditions, pursuit of changes in states of consciousness, and “to get high” (1, 13). According to a recent systematic review (13), one of the most predictive factors associated with gabapentinoid use was the concomitant use of opioids.

The neurobiological mechanism involved in the abuse liability of gabapentinoids has not been yet clearly investigated. The usual increase in the dopamine levels at the mesolimbic brain circuits has not been proved in preclinical studies (14, 15). Gabapentinoids have been reported to produce alcohol/gamma hydroxybutyrate (GHB)/benzodiazepine-type effects mixed with euphoria. Rates of euphoria have been reported at between 1 and 12% but this has been for therapeutic doses. Other reported effects include dissociative feeling, improved sociability, relaxation and sense of calm, and psychedelic effects (10, 16).

On the other hand, however, there are studies indicating that gabapentin could be an useful treatment for alcohol use disorder. For instance, a recent randomized controlled trial showed efficacy of gabapentin in the treatment of alcohol use disorder, improving the alcohol withdrawal syndrome, reducing the heavy drinking days and more total abstinence in the group treated with 1,200 mg of gabapentin (17). Also, in a meta-analysis of seven studies, gabapentin showed efficacy in the treatment of alcohol use disorder, reducing the number of heavy drinking days (18).

In countries as United Kingdom gabapentinoids have been reclassified as Class C controlled drugs under the Misuse of Drugs Act, from 1 April 2019 (19). That means that it is illegal to dispense them without a signed prescription, but that they do not require safe custody in controlled drug cabinets. In Spain, Italy, Germany, and France, gabapentin and pregabalin are available both only under a medical prescription. Alternatively, in the USA, the Drug Enforcement Administration (DEA) classifies pregabalin as a Schedule V controlled substance, or the lowest abuse potential among controlled substances, and gabapentin as a non-controlled substance (20).

The aim of this study is to determine the prevalence of prescription gabapentinoids non-medical use and associated demographics in five European countries (France, Germany, Italy, Spain, and the United Kingdom) and to evaluate the main factors related with its misuse in Spain.

## Materials and Methods

### Design and Participants

The data were obtained from the Researched, Abuse, Diversion and Addiction Related Surveillance (RADARS®) System Survey of Non-Medical Use of Prescription Drugs (NMURx) Program that collects data on respondent demographics and the prevalence, reasons of use, routes of administration, and method of drug acquisition for NMU of prescription drugs across multiple countries. The methodology and the validity of this program is explained in its validation study (21, 22).

The whole program collects information from France, Germany, Italy, Spain, and the United Kingdom. Recruitment and data collection are delivered to country-based members through a global survey panel company, in the native language of the country where the survey is undertaken and in English. Each launch has a “soft launch” of around 500 participants to ensure proper data collection. The surveys were available during the following timeframes: In France: from 2017 December 13 to 2018, January, 7: in Germany: from 2017 December 12 to 2018, January, 16; in Italy: 2017, from December 14 to December 26; in Spain: From 2017 December 12 to 2018, January, 4; and in UK: 2017, from September 28 to December 1.

The inclusion criteria were: agree to be included and give informed consent at the beginning of the survey; adult age that was defined as ages 15–110 years in Spain, 16–110 years in the United Kingdom, and 18–110 in France, Germany, and Italy; in order to reflect the geographical and gender distribution of the country, surveys from different countries and regions have been included if region/sex sampling strata that has not yet met its sampling quota; and have completed the survey in its entirety. Respondents and/or surveys were excluded from the analysis if the respondent met criteria for careless response as defined by the validation study (21).

Calibration weights were applied to the survey population to be representative of the distribution of the adult population of each of the countries included in the study based on geographic region, age, sex, limitations in daily activities, and smoking status (21). National data utilized for this weighting scheme was calculated from estimates from Eurostat and the European Social Survey; NMURx was approved by the Colorado Multiple Institutional Review Board (Protocol Number: 13-2394) and locally by the Parc de Salut Mar Ethics Committee (Protocol Number: 2017/7331/I). Data used in this analysis is from the surveys launched in the second half of 2017 (17Q4).

### Measures

Respondents were asked if they had ever used prescription gabapentin or pregabalin for any reason in their lifetime; a “yes” response classified lifetime use. If respondents reported lifetime use, they were asked about last 12- month use and last 12-month NMU, where NMU was defined as “in a way not directed by your healthcare provider.”

Basic demographics (age and gender) were collated together with data on prevalence of last 12-month gabapentin/pregabalin use and NMU.

### Analyses

The weighted proportion and 95% confidence intervals (CIs) of select demographic and respondent characteristics were calculated to describe the population. Weighted prevalence estimates and 95% CIs were calculated for last 12-month use and NMU of prescription gabapentin only, pregabalin only, and pregabalin and gabapentin. The prevalence of prescription or NMU in the last 12 months was estimated by gender and age. Differences in prevalence of prescription and were compared by gender and age range (18–24, 25–34, 35–44, 45–54, 55–64, 65+ years). Analyses were conducted in SPSS Version 25.0 (Armonk, NY).

## Results

### Survey Termination and Completion for the Five Countries

In the last quarter of 2017, approximately 63,450 French panelists were invited to participate in the survey. Of the 16,903 who initiated the survey, the inclusion and exclusion criteria below were applied and a total of 10,072 respondents were included in the analysis (5,058 (50.2%) females, 46.8±15.17 years).

In Germany, ~64,982 German panelists were invited to participate in the survey. Of the 21,977 who initiated the survey, 15,051 completed it and fulfilled the inclusion criteria (7,531 (50.0%) female, mean age 46.8 ± 14.24 years).

In Italy, 41,167 Italian panelists were invited to participate in the survey. Of the 12,766 who initiated the survey, the inclusion and exclusion criteria below were applied and 10,019 surveys were included (5,019 females (50.1%), mean age 43.5 ± 13.72 years).

**Figure 1:**
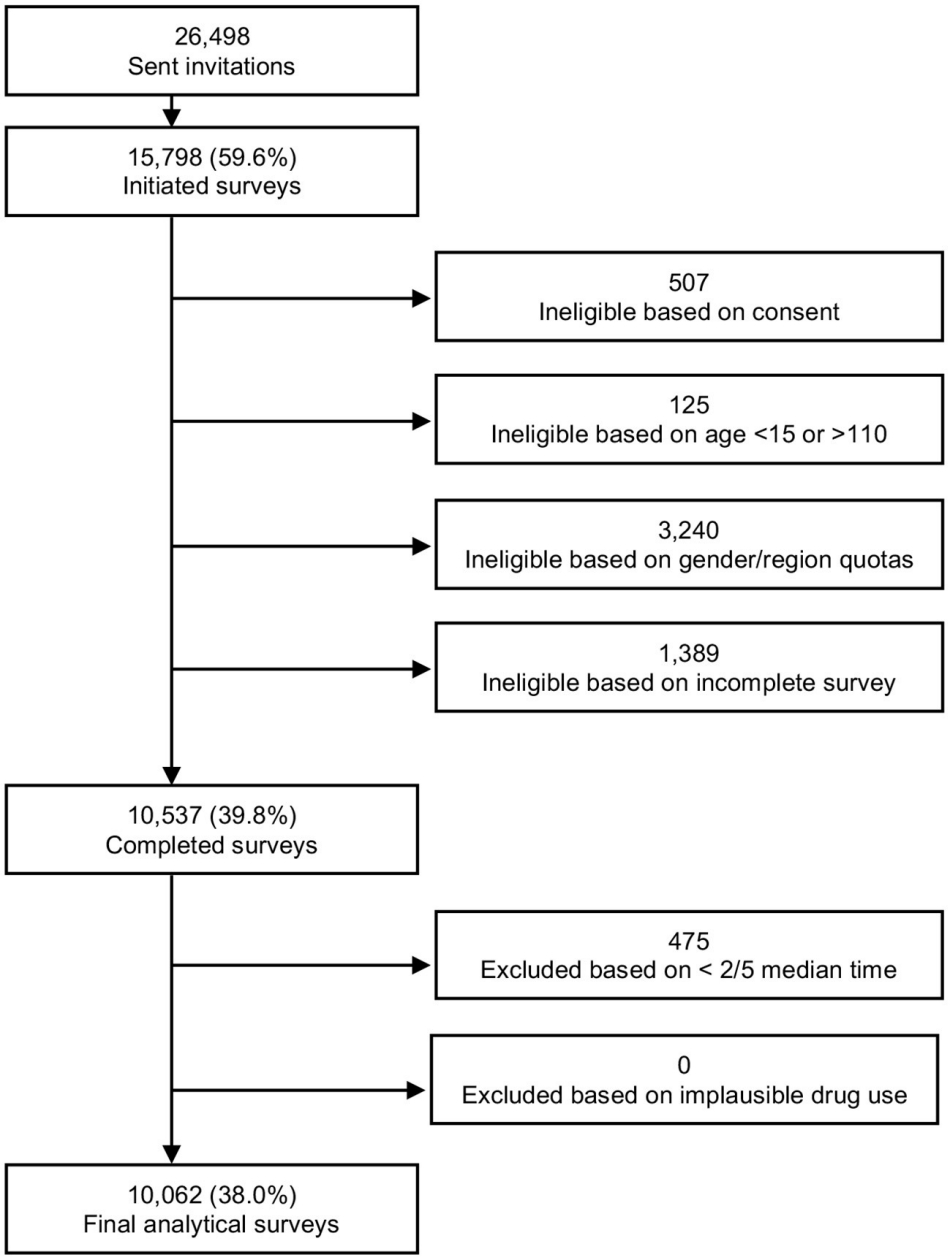
Final analytic sample flowchart.

In Spain, 26,498 panelists were invited to participate in the survey. Of the 15,798 who initiated the survey, the inclusion and exclusion criteria below were applied (Figure 1). Finally, 10,062 people completed the survey (5,030 (50.0%) female, mean age: 41.6 ± 12.74 years).

In the United Kingdom, there were 108,633 panelists invited to participate in the survey, of which 13,036 initiated the survey and 10,004 were included in the analysis (5,003 (50.0%) females, mean age 51.6 ± 15.33 years).

### Comparison of Five Countries

Prevalence of past 90 day GABA analog NMU was highest in Germany (1,191.7 per 100,000 population, 95% CI 1,004.3–1,379.1) and the UK (1,067.2, 95% CI 851.3–1,283.2), and lowest in Spain (344.4, 95% CI 204.8–484.0) and Italy (366.2, 95% CI 207.7–524.6) (Table 1).

**Table 1:** Last 90 day non-medical use of GABA analogs by country.

	**France**	**Germany**	**Italy**	**Spain**	**UK**
**Rate (95% CI) per 100,000 Adult Population**^***a***^	574.2 (424.4, 724.0)	1191.7 (1004.3, 1379.1)	366.2 (207.7, 524.6)	344.4 (204.8, 484.0)	1067.2 (851.3, 1283.2)
**Rate (95% CI) per 100,000 Standard Units**^***b***^	216.8 (160.2, 273.4)	470.4 (396.5, 544.4)	242.6 (137.6, 347.6)	105.9 (63.0, 148.9)	174.0 (138.8, 209.2)

a *Rates based on the weighted estimated number of adults who reported NMU of each drug class in the last 90 days per 100,000 adult population*.

b *Rates are based on the weighted estimated number of adults who reported NMU of each drug class in the last 90 days per 100,000 standard units sold*.

NMU was evenly distributed between genders except in Spain which showed a male predominance (Table 2). Those who non-medically use GABA analogs were estimated to have higher incidence of lifetime chronic pain, lifetime illicit drug use, and previous substance abuse therapy (Table 2).

**Table 2:** Demographics of those who have non-medically used GABA Analogs in the last 90 days vs. the general adult population demographics.

	**France**		**Germany**		**Italy**		**Spain**		**UK**	
	**GABA analog NMU**	**General population**	**GABA analog NMU**	**General population**	**GABA analog NMU**	**General population**	**GABA analog NMU**	**General population**	**GABA analog NMU**	**General population**
**Male**	56.8% (48.19, 65.36)	47.6% (46.59, 48.68)	54.0% (48.13, 59.90)	48.6% (47.79, 49.49)	45.0% (33.54, 56.38)	49.1% (47.89, 50.21)	65.7% (53.60, 77.79)	48.6% (47.47, 49.79)	46.8% (39.82, 53.83)	48.8% (47.61, 49.94)
**Chronic pain during lifetime**	72.1% (64.39, 79.88)	33.0% (32.03, 34.05)	77.6% (72.26, 82.46)	39.2% (38.41, 40.09)	68.6% (57.86, 79.26)	29.4% (28.29, 30.47)	63.1% (51.92, 74.37)	30.9% (29.82, 32.05)	70.3% (63.79, 76.72)	38.9% (37.84, 40.04)
**Lifetime illicit drug use**	33.9% (25.71, 42.05)	18.2 (17.39, 18.94)	28.7% (23.42, 34.05)	25.4% (24.70, 26.17)	37.8% (26.47, 49.04)	20.5% (19.64, 21.38)	42.0% (30.86, 53.20)	24.4% (23.45, 25.32)	48.0% (40.95, 54.98)	27.3% (26.22, 28.31)
**Previous substance abuse treatment**	11.5% (6.04, 16.89)	1.7% (1.46, 1.97)	7.7% (4.56, 10.91)	1.8% (1.59, 2.05)	3.8% (0.00, 8.40)	0.6% (0.47, 0.80)	13.3% (6.45, 20.21)	2.2% (1.92, 2.55%)	19.3% (13.35, 25.21)	1.7% (1.41, 2.02)

### Spanish Respondents Characteristics

Approximately 26,498 Spanish panelists were invited to participate in the survey. Of the 15,798 who initiated the survey, the inclusion and exclusion criteria below were applied (Figure 1). Finally, 10,062 people completed the survey (5,030 (50.0%) female, mean age: 41.6 + 12.74 years). The main characteristics (unweighted and weighted) of the respondents are described in Table 3. The responses are weighted to represent the population above 15 years old in Spain by region, gender and age.

**Table 3:** Spanish survey respondents' demographics (*N* = 10,062).

**Variable**	**Unweighted** ***N* (%)**	**Weighted^**a**^** **% (95% CI)**
**Gender**		
Male	5,032 (50.0%)	48.8 (47.6, 49.9)
**Age (years)**		
Mean (STD)	41.6 (12.74)	45.7 (0.2)
Median (IQR)	41.0 (32.0, 50.0)	46.0 (33.6, 56.9)
Range	(15.0, 90.0)	(15.0, 90.0)
**Age categories (years)**		
15–24	1,008 (10.0%)	0 (0.0, 0.0)
25–34	2,021 (20.1%)	0 (0.0, 0.0)
35–44	2,999 (29.8%)	14.4 (13.8, 15.0)
45–54	2,391 (23.8%)	19.8 (19.0, 20.5)
55+	1,643 (16.3%)	18.2 (17.5, 19.0)
**Territory of residence**		
Andalucía	1,813 (18.0%)	18.0 (17.1, 18.9)
Aragón	570 (5.7%)	5.7 (5.2, 6.3)
Canarias	467 (4.6%)	4.7 (4.2, 5.1)
Cantabria	51 (0.5%)	0.5 (0.3, 0.7)
Castilla y León	617 (6.1%)	6.1 (5.5, 6.7)
Castilla-La Mancha	424 (4.2%)	4.1 (3.6, 4.6)
Cataluña	1,673 (16.6%)	17.1 (16.3, 18.0)
Ciudad Autónoma de Ceuta	2 (0.0%)	0.0 (0.0, 0.0)
Ciudad Autónoma de Melilla	10 (0.1%)	0.2 (0.1, 0.3)
Comunidad de Madrid	1,380 (13.7%)	13.7 (12.9, 14.5)
Comunidad Foral de Navarra	65 (0.6%)	0.6 (0.5, 0.8)
Comunidad Valenciana	1,105 (11.0%)	10.4 (9.8, 11.1)
Extremadura	188 (1.9%)	1.9 (1.5, 2.2)
Galicia	643 (6.4%)	6.2 (5.6, 6.7)
Illes Balears	131 (1.3%)	1.3 (1.1, 1.6)
La Rioja	39 (0.4%)	0.4 (0.2, 0.5)
País Vasco	290 (2.9%)	2.9 (2.5, 3.3)
Principado de Asturias	283 (2.8%)	3.0 (2.6, 3.5)
Región de Murcia	311 (3.1%)	3.1 (2.7, 3.5)
**Region of residence**		
Noroeste	977 (9.7%)	9.7 (9.0, 10.5)
Noreste	964 (9.6%)	9.6 (9.0, 10.3)
Comunidad de Madrid	1,380 (13.7%)	13.7 (12.9, 14.5)
Centro	1,229 (12.2%)	12.1 (11.3, 12.9)
Este	2,909 (28.9%)	28.9 (27.9, 29.9)
Sur	2,136 (21.2%)	21.3 (20.3, 22.2)
Canarias	467 (4.6%)	4.7 (4.2, 5.1)
**Net monthly household income**		
Under €499	404 (4.0%)	4.0 (3.5, 4.4)
Between €500 and €799	430 (4.3%)	4.6 (4.1, 5.1)
Between €800 and €999	588 (5.8%)	5.9 (5.3, 6.4)
Between €1.000 and €1.499	2,145 (21.3%)	20.5 (19.6, 21.4)
Between €1.500 and €1.999	1,723 (17.1%)	16.6 (15.8, 17.5)
Between €2.000 and €2.499	1,472 (14.6%)	14.4 (13.5, 15.2)
Between €2.500 and €2.999	1,105 (11.0%)	11.1 (10.4, 11.9)
Between €3.000 and €4.999	1,116 (11.1%)	11.5 (10.7, 12.2)
Between €5.000 and €6.999	204 (2.0%)	2.1 (1.8, 2.5)
€7.000 or more	92 (0.9%)	0.8 (0.7, 1.0)
Prefer not to say	783 (7.8%)	8.5 (7.8, 9.2)
**Marital status**		
Single	3,709 (36.9%)	32.3 (31.3, 33.3)
Married	5,463 (54.3%)	55.5 (54.3, 56.6)
Separated/divorced	760 (7.6%)	9.5 (8.7, 10.3)
Widowed	130 (1.3%)	2.7 (2.2, 3.2)
**Education achieved**		
No studies or incomplete primary studies	25 (0.2%)	0.3 (0.2, 0.5)
Comprehensive primary education	174 (1.7%)	2.3 (1.9, 2.7)
Secondary studies 1st stage	1,481 (14.7%)	15.8 (14.9, 16.7)
Secondary studies 2nd stage	3,544 (35.2%)	35.9 (34.8, 37.0)
Middle University studies	2,274 (22.6%)	22.0 (21.0, 22.9)
Higher University studies	2,564 (25.5%)	23.7 (22.7, 24.6)
**Student within the last 3 months**		
Yes	1,403 (13.9%)	13.6 (12.8, 14.3)
No	8,659 (86.1%)	86.4 (85.7, 87.2)
**A member or former member of the armed forces**		
Yes	479 (4.8%)	4.6 (4.2, 5.1)
No	9,583 (95.2%)	95.4 (94.9, 95.8)
**Currently a healthcare professional**		
Yes	615 (6.1%)	5.6 (5.1, 6.1)
No	9,447 (93.9%)	94.4 (93.9, 94.9)
**Pregnancy status**^**b**^		
Yes	250 (5.0%)	3.3 (2.9, 3.8)
No	4,780 (95.0%)	96.7 (96.2, 97.1)
**Gestation**^**c**^ **(months)**		
Mean (STD)	4.8 (2.08)	4.8 (0.1)
Median (IQR)	5.0 (3.0,6.0)	4.4 (2.6,5.9)
Range	(1.0, 9.0)	(1.0, 9.0)
**Survey language**		
English	197 (2.0%)	2.1 (1.8, 2.5)
Spanish	9,865 (98.0%)	97.9 (97.5, 98.2)

*CI, Confidence Interval; STD, Standard deviation; IQR, Interquartile range*.

a *Responses are weighted to represent the distribution of adults (ages 15+) in Spain by region, gender, and age*.

b *Among females only (n = 5,030)*.

c *Among pregnant females only (n = 250)*.

A total of 1,003 (10.0%) respondents referred a lifetime use of gabapentinoids; after weighting the responses a 9.9% (95% CI: 9.2–10.6) (Table 4).

**Table 4:** Respondents that reported use of gabapentinoids (from total survey respondents *n* = 10,062).

	**Unweighted *N* (%)**	**Weighted^**a**^ % (95% CI)**
Lifetime use	1,003 (10.0%)	9.9 (9.2, 10.6)
Lifetime non-medical use	323 (3.2%)	2.9 (2.6, 3.3)
Last 12 month non-medical use	169 (1.7%)	1.5 (1.2, 1.7)
Last 90 day non-medical use	45 (0.4%)	0.4 (0.3, 0.6)
Last 30 day non-medical use	42 (0.4%)	0.4 (0.3, 0.6)
Last 7 day non-medical use	35 (0.3%)	0.4 (0.2, 0.5)

*CI, Confidence Interval*.

a *Responses are weighted to represent the distribution of adults (ages 15+) in Spain by region, gender, and age*.

From the total Spanish sample, 444 (4.4%) respondents have ever used gabapentin and 624 (6.2%) pregabalin. Out of them, 84 (18.9%), and 126 (20.6%) reported non-medical use of gabapentin and pregabalin, respectively (cave: according to Table 4 the % of respondents with NMU of gabapentinoids should be something higher >>2.9 out of 9.9.% = 29.3%. The others respondents were not sure (40 (9.0%) for gabapentin and 33 (5.3%) for pregabalin) about their NMU (that means, that they were not sure whether they followed the recommendations of the prescriber) or answered that they do not use for NMU (320 (72.1%) for gabapentin and 465 (74.5%) for pregabalin).

### Characteristics of Non-medical Use in Spain

The main reasons for non-medical use were to self-treat pain and other medical condition different from pain (Table 5).

**Table 5:** Reasons for non-medical use in the Spanish sample.

	***N*^**a**^**	**To self-treat my pain *N* (%)**	**To treat a medical condition, other than pain *N* (%)**	**For enjoyment to get high *N* (%)**	**To come down *N* (%)**	**To prevent or treat withdrawal symptoms *N* (%)**	**Other reason *N* (%)**
Gabapentin	124	65 (52.4%)	40 (32.3%)	12 (9.7%)	10 (8.1%)	13 (10.5%)	20 (16.1%)
Pregabalin	159	76 (47.8%)	45 (28.3%)	14 (8.8%)	9 (5.7%)	9 (5.7%)	28 (17.6%)

a *Includes all survey respondents who report non-medical use of the product*.

*Respondents may check multiple options, percentages may not sum to 100*.

Respondents who declare NMU of gabapentinoids, usually used the oral route of administration (either swallowed or chewed and then swallowed). Those of them who used to get high, reported to inject gabapentin (41%) and pregabalin (14.3%) (Table 6).

**Table 6:** Route of administration by reason for non-medical use in Spain.

**Reason for NMU**	***N*^**a**^**	**Swallowed *N* (%)**	**Chewed and then swallowed *N* (%)**	**Dissolved in mouth (e.g., between cheek and gum, under tongue) *N* (%)**	**Inhaled (snorted or smoked) *N* (%)**	**Injected (shot it up) *N* (%)**	**Other route *N* (%)**
**Gabapentin (e.g., Gabatur, Neurontin®, or generic), tablets/capsules**							
To self-treat my pain	65	47 (72.3%)	24 (36.9%)	19 (29.2%)	13 (20.0%)	12 (18.5%)	10 (15.4%)
To treat a medical condition, other than pain	40	27 (67.5%)	13 (32.5%)	14 (35.0%)	7 (17.5%)	9 (22.5%)	3 (7.5%)
For enjoyment/to get high	12	4 (33.3%)	5 (41.7%)	3 (25.0%)	1 (8.3%)	5 (41.7%)	2 (16.7%)
To come down	10	3 (30.0%)	5 (50.0%)	6 (60.0%)	1 (10.0%)	3 (30.0%)	0 (0.0%)
To prevent or treat withdrawal symptoms	13	5 (38.5%)	6 (46.2%)	7 (53.8%)	6 (46.2%)	4 (30.8%)	4 (30.8%)
Other reason	20	10 (50.0%)	6 (30.0%)	5 (25.0%)	6 (30.0%)	3 (15.0%)	5 (25.0%)
**Pregabalin (e.g., Lyrica® or generic), tablets/capsules**							
To self-treat my pain	76	53 (69.7%)	19 (25.0%)	13 (17.1%)	12 (15.8%)	8 (10.5%)	5 (6.6%)
To treat a medical condition, other than pain	45	30 (66.7%)	21 (46.7%)	8 (17.8%)	4 (8.9%)	5 (11.1%)	1 (2.2%)
For enjoyment/to get high	14	2 (14.3%)	6 (42.9%)	8 (57.1%)	4 (28.6%)	2 (14.3%)	1 (7.1%)
To come down	9	1 (11.1%)	7 (77.8%)	3 (33.3%)	3 (33.3%)	2 (22.2%)	1 (11.1%)
To prevent or treat withdrawal symptoms	9	5 (55.6%)	6 (66.7%)	4 (44.4%)	4 (44.4%)	3 (33.3%)	1 (11.1%)
Other reason	28	17 (60.7%)	9 (32.1%)	7 (25.0%)	5 (17.9%)	4 (14.3%)	10 (35.7%)

a *Includes all survey respondents who report each reason for non-medical use of the product*. *Respondents may check multiple options, percentages may not sum to 100*.

Respondents said that they main method of drug acquisition in Spain was by a prescription of a doctor/dentist (61.3% for gabapentin and 69.8% for pregabalin), however, they used several methods to acquire them including family or friends (either bought or given), taken from family, friends and other people, bought outside the country, by internet or to a dealer (Table 7).

**Table 7:** Reported method of drug acquisition in Spain.

	***N*^**a**^**	**Was prescribed it by a doctor or dentist** ***N* (%)**	**Bought it or was given it by friends or family members** ***N* (%)**	**Took it from friends or family members without their knowledge** ***N* (%)**	**Took it from someone other than friends/ family without their knowledge** ***N* (%)**	**Bought it abroad (outside Spain) without a Rx** ***N* (%)**	**Bought it on the internet without a Rx** ***N* (%)**	**Bought it from a dealer** ***N* (%)**
Gabapentin	124	76 (61.3%)	35 (28.2%)	30 (24.2%)	39 (31.5%)	33 (26.6%)	32 (25.8%)	38 (30.6%)
Pregabalin	159	111 (69.8%)	51 (32.1%)	40 (25.2%)	33 (20.8%)	41 (25.8%)	39 (24.5%)	46 (28.9%)

a *Includes all survey respondents who report non-medical use of the product*.

*Respondents may check multiple options, percentages may not sum to 100*.

Finally, in Table 8, is described the last purchase of gabapentin and pregabalin where respondents said that they have obtained the substances from a dealer of bought in internet. The median price paid for both was similar (10 €).

**Table 8:** Last purchase characteristics in Spain.

	***N*^**a**^**	**Number/volume purchased**	**Strength^**b**^**	**Total price paid (€)**
Gabapentin	45	N: 45 Mean (STD): 5.8 (8.53) Median (IQR): 2.0 (1.0, 6.0) Range: (0.0, 33.0)	N: 12 Mean (STD): 38.9 (49.44) Median (IQR): 8.0 (2.0, 100.0) Range: (1.0, 120.0)	N: 45 Mean (STD): 14.4 (15.42) Median (IQR): 10.0 (2.0, 20.0) Range: (0.0, 55.0)
Pregabalin	52	N: 52 Mean (STD): 5.4 (7.65) Median (IQR): 2.0 (1.0, 6.0) Range: (0.0, 35.0)	N: 16 Mean (STD): 26.3 (61.40) Median (IQR): 3.5 (2.0, 27.0) Range: (1.0, 250.0)	N: 52 Mean (STD): 185,204.8 (1,302,466.10) Median (IQR): 10.0 (2.0, 32.0) Range: (0.0, 9,393,939.0)

*STD, Standard deviation; IQR, Interquartile Range*.

a *Includes all survey respondents who report non-medical use of the product and “Bought it from a dealer” or “Bought it on the internet”*.

b *Strength: MG per tablet/capsule, MCG/h per patch, MG per oral film, MG/ML per liquid, MCG per lollipop, MCG per lozenge, MG per suppository; All non-numeric entries were excluded*.

*Respondents have option to check ‘I'm not sure' under strength*.

## Discussion

The main finding of this study is that it confirms the potential abuse liability and then non-medical use of the gabapentinoids gabapentin and pregabalin. When comparing the five European countries, those who non-medically use gabapentinoids were estimated to have a higher likelihood of chronic pain, use of illicit substances, and history of substance abuse treatment compared to the general population. These results are in concordance with country surveys, reviews and metanalyses published previously (8, 23, 24).

There are differences in the rate per 100,000 people among the five countries, with Germany and UK the countries having a higher rate compared to France, Italy and Spain. Reasons for these differences could be related to the availability of other sedative type substances in those countries. According to the European Drug Report of the same year that the information of this study was recorded (25), the prevalence of cannabis use in France, Italy, and Spain was higher than 15%, whereas, in Germany and United Kingdom the prevalence was lower than 15%. We can hypothesize that some reasons for using cannabis and gabapentinoids could be similar: to treat pain and anxiety symptoms; in countries with higher availability of cannabinoids and opioids, subjects could prefer them to gabapentinoids. Also, in some countries, gabapentinoids might replace partially benzodiazepines; in Spain, a general population survey performed every 2 years, showed data on life-time NMU of benzodiazepines about 3.0% in male and 3.1% in female (26). Some studies have tried to analyze the possible usefulness of pregabalin and gabapentin in the treatment of benzodiazepine use disorder, but there are no clear results regarding this (27, 28).

When evaluating the rates by drug, as described before, pregabalin has more endorsements than gabapentin, for example, in a recent paper describing data from addictovigilance monitoring for gabapentinoids (24). Some publications have described a higher abuse liability for pregabalin compared to gabapentin. One of the explanations of this difference could be the higher prevalence of euphoria in pregabalin compared to gabapentin. The studies that have described this effect reported that this is a dose-dependent effect and it is not related to treatment indication, nor previous abuse of substances; its prevalence varies among different studies from 1 to 40% (10, 29). The theory of people taking pregabalin to experience euphoria and to get high it is not completely explained by our results, as the majority of the respondents used pregabalin as self-treatment. The differences between the two substances could also be explained by the different pharmacokinetic characteristics of both molecules; pregabalin has more rapid absorption than gabapentin; also, the peak plasma concentration is more rapidly achieved with pregabalin (1 h compared to 4–5 h) and has a longer half-life (7).

In the subsample of Spanish population evaluated, about 20% of all persons ever using gabapentinoids report on NMU of these substances. A risk for NMU that should not be neglected. The main reason for non-medical use was in both medications for self-treat any pain, followed to treat other medical conditions; few respondents used them to get high or to come down; also, there were a percentage of people using them to prevent withdrawal symptoms. Another article, based on data of pharmavigilance (24), found that the use of pregabalin was not only related to the objective to get high, but also, to prevent withdrawal symptoms, as a substitute of other substances and to potentiate the effect of other drugs (mainly benzodiazepines and opioids). In our sample, the inhaled and intravenous route were mainly reported for those who use pregabalin and gabapentin to prevent withdrawal syndrome, to come down and to get high. It is important to consider the possibility of using the intravenous route, and asking patients about it to prevent the transmission of blood borne infections (Hepatitis B and C, and HIV).

Another aspect to take into account may be the polymedication risk. Pregabalin and gabapentin are usually prescribed with other pain medications, mainly with opioids; among 50–70% were reported in a recent paper (23). This combination could increase the risk for overdose death (30). Otherwise, the usefulness of the combination of pregabalin and opioids for the treatment of some kind of pain is not clear, as some researchers have described that pregabalin plus opioids was associated with more pain severity and higher oral doses of opioids; furthermore, pregabalin use was not associated with improvements on mental health symptoms (31).

When prescribing these medications it is important to be aware and monitor for signs of misuse and overdosification, mainly in patients with risk factors for NMU (previous history of substance use disorder and chronic pain). It is important to remark that, although NMU of gabapentinoids is more frequent in patients with previous substance use disorder, there are described cases of a primary abuse in people without any of the known risk factors (24), for this reason, it is important to monitor for signs of NMU in all patients in treatment with gabapentinoids. The detection of NMU could be complicated as these medications are not detected in routine toxicology urine controls. Furthermore, prescribers should be aware of the risk of NMU, when patients request for specific drugs of higher doses, when they obtain medications from different sources (doctor shopping), when the medications are lost or stolen frequently or they ask for new prescriptions too early (1).

The NMURx survey methodology is useful to identify under-documented use and misuse of medication and can detect changes in trends of substance use and misuse; also, it permits to make comparisons among different countries. The large sample size and post-stratification weighting applied creates estimates that are representative of general populations. However, there are some limitations related to online surveys, in first place the reliance of participants to provide honest responses; also, another limitation of the study is that respondents who have acquired a gabapentinoid product from a family member, friend, or dealer may not be aware whether it was initially obtained with a prescription or from another source. However, these limitations will apply to all surveys so still allow for comparison across countries.

In conclusion, in spite of the risk of NMU, gabapentinoids are useful medications in the treatment of neuropathic pain, generalized anxiety disorder, and some forms of epilepsy. Professionals prescribing these medications should be aware and actively search for signs of misuse and diversion.

## Data Availability Statement

The raw data supporting the conclusions of this article will be made available by the authors, without undue reservation.

## Ethics Statement

The studies involving human participants were reviewed and approved by the Colorado Multiple Review Board (Protocol Number: 13-2394) and locally by the Parc de Salut Mar Ethics Committee (Protocol Number: 2017/7331/I). Written informed consent to participate in this study was provided by the participants' legal guardian/next of kin.

## Author Contributions

FF, PD, DW, NS, MG, IM, MA, and RD were responsible to prepare and adapt the country protocols. MG and RD were responsible for the project concept and study design. FF, WL, EP, and MF contributed to drafting the manuscript. MF, NS, and MT were responsible for the final revision. All authors have read and approved the final submitted manuscript.

## Conflict of Interest

FF has received during the last 3 years travel grants from Lundbeck, Otsuka, Indivior, Pfizer, Gilead, Angelini, and Servier; and she has received grant/research support from Indivior and Servier. MT has been consultant/advisor and/or speaker for Gilead Sciences, Merck Sharp and Dohme Corp, Indivior, Mundipharma Pharmaceutics, Servier, and Adamed. NS received honoraria for several activities (advisory boards, lectures, manuscripts) by the factories AbbVie, Camurus, Hexal, Janssen-Cilag, MSD, Medice, Mundipharma, Reckitt-Benckiser/Indivior, and Sanofi-Aventis. During the last 3 years he participated in clinical trials financed by the pharmaceutical industry. IM served as Board Member for Angelini, Camurus, CT Sanremo, D&A Pharma, Gilead, Indivior, Lundbeck, Molteni, MSD, and Mundipharma. MA over the past 3 years has interacted directly or through the University of Bordeaux Foundation with Camurus, Mundipharma, Accord Healthcare, Indivior for expert advice and/or funding donation grants. The remaining authors declare that conflicts of interest had no role in the design of the study, data collection, analyses, and interpretation, in the writing of the manuscript, or in the decision to publish the results.
